# Antimicrobial photodynamic therapy: an effective alternative approach to control fungal infections

**DOI:** 10.3389/fmicb.2015.00202

**Published:** 2015-03-13

**Authors:** Ludmila M. Baltazar, Anjana Ray, Daniel A. Santos, Patrícia S. Cisalpino, Adam J. Friedman, Joshua D. Nosanchuk

**Affiliations:** ^1^Department of Microbiology and Immunology, Albert Einstein College of Medicine, BronxNY, USA; ^2^Department of Medicine, Albert Einstein College of Medicine, BronxNY, USA; ^3^Departamento de Microbiologia, Instituto de Ciências Biológicas, Universidade Federal de Minas GeraisBelo Horizonte, Brazil; ^4^Division of Dermatology, Department of Medicine, Albert Einstein College of Medicine, BronxNY, USA; ^5^Department of Physiology and Biophysics, Albert Einstein College of Medicine, BronxNY, USA

**Keywords:** photodynamic inhibition, fungal cells, treatment, photosensitizer, light source, photochemicals and photobiological events

## Abstract

Skin mycoses are caused mainly by dermatophytes, which are fungal species that primarily infect areas rich in keratin such as hair, nails, and skin. Significantly, there are increasing rates of antimicrobial resistance among dermatophytes, especially for *Trichophyton rubrum*, the most frequent etiologic agent worldwide. Hence, investigators have been developing new therapeutic approaches, including photodynamic treatment. Photodynamic therapy (PDT) utilizes a photosensitive substance activated by a light source of a specific wavelength. The photoactivation induces cascades of photochemicals and photobiological events that cause irreversible changes in the exposed cells. Although photodynamic approaches are well established experimentally for the treatment of certain cutaneous infections, there is limited information about its mechanism of action for specific pathogens as well as the risks to healthy tissues. In this work, we have conducted a comprehensive review of the current knowledge of PDT as it specifically applies to fungal diseases. The data to date suggests that photodynamic treatment approaches hold great promise for combating certain fungal pathogens, particularly dermatophytes.

## Introduction

Fungi are eukaryotic organisms and their similarities to mammalian cells have led to significant difficulties in the development of new antifungal drugs. Fungal infections are an important health problem worldwide, affecting both immunocompetent and immunocompromised individuals. Acquisition of fungal pathogens results in varied outcomes ranging from asymptomatic infection to rapidly lethal systemic disease (Cowen, 2008).

Though cutaneous mycoses are rarely life-threatening; they result in significant morbidity, causing discomfort, disfigurement, social isolation, and may predispose to bacterial diseases (Brown et al., 2012). These mycoses are frequently recurrent and chronic. Moreover, they are extremely common as it is estimated that 10–20% of the worldwide population may be affected (Drake et al., 1996; El-Gohary et al., 2014). The main fungal skin diseases are caused by *Malassezia* sp. and the dermatophytes (White et al., 2014).

*Malassezia* sp. are frequent commensal inhabitants of the skin and scalp that can cause a range of diseases, including pityriasis versicolor, dandruff, and seborrheic dermatitis (Gaitanis et al., 2013). These agents are associated with ∼50% of the dermal disorders in healthy humans and 70–75% of immunosuppressed individuals (White et al., 2014). The dermatophytes are a group of filamentous fungi that are the etiologic agent of dermatophytosis, diseases affecting skin, hair, and nails. The dermatophytes produce enzymes that digest keratin, which the fungi use as a food source, but also facilities their capacity to infect tissues containing keratin (Weitzman and Summerbell, 1995). Immunocompromised individuals are at increased risk for dermatophytoses, including progression to disseminated disease (Nenoff et al., 2014b). Although clinical resistance to current antifungal drugs has been well documented, clinical failures are most often associated with discontinuation of the treatment by the patient (Mukherjee et al., 2003). Although some diseases caused by *Malassezia* sp. and the dermatophytes can be eradicated with several days of antifungal therapy, months of therapy may be required for combating infections of the nails or diseases in the setting of immune deficiency. Typically administered antifungal drugs include azoles, allylamines, ciclopirox, and amorolfine (Gupta and Cooper, 2008).

Photodynamic therapy (PDT) is an alternative approach to these antifungal medications that primarily target ergosterol production. Antimicrobial photodynamic inhibition (aPI) or therapy (aPDT) combines a pharmacologically inert chromophore, termed a photosensitizer (PS), with a light corresponding to the chromophore’s specific absorption wavelength (Dai et al., 2012). This exposure of the chromophore to the specific light wavelength induces the production of harmful radicals, such as reactive species of oxygen (ROS) and nitrogen (RNS), which are capable of killing cells (Hamblin and Hasan, 2004). The ability of aPI to kill microbes has been described by several investigators, and the data suggests that aPI is potentially effective against bacterial, viral, fungal, and protozoal infections (reviewed in Hamblin and Hasan, 2004; Krausz and Friedman, 2014). Significantly, investigators have shown that aPDT effectively inactivates *Trichophyton rubrum*, the most common causative agent of dermatophytosis (Smijs and Pavel, 2011; Nenoff et al., 2014a). In this review, we provide a detailed overview of the promise of aPDT in the context of the fungal infections, describing *in vitro*, preclinical, and human studies.

## Brief History of Photodynamic Therapy

The use of light combined with a photosensitive substance is actually an ancient approach for the treatment of skin diseases. There are documents from ∼1200–2000 BC showing that Egyptian and Chinese physicians as well as Indian Hindu Ayurvedic practitioners used combinations of plant extracts with exposure to sunlight to treat skin disorders (Pathak and Fitzpatrick, 1992; Craig et al., 2014). For example, the Egyptians used the application of an extract of *Ammi majus*, a furanocoumarin-containing plant, associated with sun exposure to topically treat vitiligo. In Ayurvedic traditional medicine, an extract of *Psoralea corylifolia*, which is a furanocoumarin, was similarly used for vitiligo (Pathak and Fitzpatrick, 1992).

However, the term PDT was coined in 1900 by Tappeiner and his co-workers in Germany (Tappeiner, 1900). The first detailed report of the observation that the combination of light and dye could be harmful to a cell was published by Raab (1900), a student of Tappeiner. Rabb observed that the protozoon *Paramecium caudatum* died after light exposure in the presence of an acridine dye and the amount of light exposure correlated with the killing efficiency of the system. Following this finding, in 1903, Tappeiner and the dermatologist Jesionek translated their findings from the bench to the bedside in a report that detailed how the topical application of eosin associated with exposure to white light effectively treated a skin tumor (Jesionek and Tappeiner, 1903). Significantly, Tappeiner and his colleague Jodlbauer also noted that the phototoxic effect did not occur in the absence of oxygen and they introduced the term “photodynamic action” in 1907 to describe this reaction (Tappeiner and Jodlbauer, 1904, 1907).

The first PS broadly used in the medicine was the porphyrin hematoporphyrin (Hp), obtained from dried blood after treatment with concentrated sulfuric acid. Hp was first tested *in vitro* by Hausman (1911) in Austria, where he demonstrated that the activated compound was effective against paramecia and erythrocytes (Scherer, 1841). Hausman (1911) described the phototoxic effect of Hp on murine skin after systemic application of Hp followed by exposing the mice to light (Hausman, 1911). Meyer-Betz (1913) pioneered the study of Hp as PS in humans when he self-injected 200 mg of Hp. After exposure to sunlight, Meyer–Betz suffered a painful phototoxic reaction that lasted for over 2 months. Policard (1924) described the affinity of endogenous porphyrins to tumors by using a Wood lamp to detect a red fluorescence in rat sarcoma after Hp application. Schwartz et al. (1955) in the USA, demonstrated that the phototoxic effect of Hp could be reduced by treatment of Hp with acetic acid and sulfuric acid, obtaining a mixture of porphyrin, a hematoporphyrin derivate (HpD). This improved photosensitizing compound had high affinity for tumors and the ability to detect tumors using HpD was demonstrated by Lipson and Baldes (1960). The first systematic trial of PDT was reported by Dougherty et al. (1978), in which 113 cutaneous and subcutaneous tumors were subjected to HpD and red light resulting in 111 partial or total responses to therapy. The first PS to gain federal approval for clinical use was Photofrin^®^ in Canada in 1993, and other countries subsequently followed, including the U.S. Food and Drug Administration (FDA) in 1995 (Usuda et al., 2006). With the broader entry of PDT into clinical practice as a chemotherapeutic modality, investigators have increasingly explored PDT as an alternative approach to combat infectious diseases.

## Photosensitizer and Light Properties

Photosensitizers are dyes with the capacity of absorb energy from a light source and transfer this energy to another molecule (Plaetzer et al., 2009). An effective PS is typically characterized by water solubility, minimal dark toxicity, a low mutagenic potential, and highly chemically stable. The PS should have the ability to accumulate preferentially in the specific tissue/cell target and be rapidly eliminated after administration to avoid prolonged photosensitization (Nyman and Hynninen, 2004; Plaetzer et al., 2009). In addition, the waveband of absorption of the PS should be between 600 and 800 nm in order to avoid skin phototoxicity. This therapeutic window minimizes (1) absorption during exposure to typical daylight (wavelength 400–600 nm) and (2) its absorption by water molecules, which increases at wavelengths above 800 nm (Plaetzer et al., 2009; Sekkat et al., 2012). More recently, researchers have designed modern carriers such as liposomes, nanoparticles, and microspheres to reduce chromophore self-aggregation in fluid mediums and increase the selectivity of the PS (Nyman and Hynninen, 2004).

The major PSs used in modern clinical trials are the phenothiazine salts toluidine blue O (TBO) and methylene blue (MB), with wavelengths of absorption of 600–660 nm (Calzavara-Pinton et al., 2012). Both are clinically approved for human use and, notably, they can effectively act on the fungal membrane, causing structural damage (Plaetzer et al., 2009; Calzavara-Pinton et al., 2012; Dai et al., 2012). Other substances, such as porphyrins, phthalocyanines, 5-aminolevulinic acid (ALA) and curcumin, have also been used as PSs. Porphyrin dyes (absorption at 400–650 nm range), can cause alterations at cell membranes, allowing the penetration of the PS into the cell with consequent damage to intracellular targets (Cormick et al., 2009; Calzavara-Pinton et al., 2012). Phthalocyanines (absorption at 630–720 nm range) are similar to porphyrins compounds (Calzavara-Pinton et al., 2012; Sekkat et al., 2012); however, they are strongly hydrophobic, a characteristic that is usually balanced by modifications in its chemical structure to improve water solubility (Mantareva et al., 2011; Sekkat et al., 2012). ALA is not intrinsically photodynamically active, but irradiation of cells containing ALA produces a range of endogenous PS that generate reactive oxygen species (ROS), which damage mitochondria and plasma membranes (Harris and Pierpoint, 2012). Curcumin (absorption at 408–434 nm range) is a yellow dye (also known as the spice turmeric) isolated from *Curcuma longa* that is a well established PS (Dovigo et al., 2013) and PDT with curcumin generates high levels of ROS that cause cell death by apoptosis (Sharma et al., 2010).

Currently, both coherent (lasers) and non-coherent (diode emission of light – LED and lamps) light sources are used for PDT (Nyman and Hynninen, 2004; Calin and Parasca, 2009). Lasers are able to deliver light with high degrees of monochromaticity that can be focused into an optic fiber. However, the high cost and the difficulties to transport are some of the drawbacks for the use of lasers in PTD. LEDs are less expensive, easily transportable and, with the discovery of PSs with longer wavelengths, are increasingly being used in experimental and clinical applications of PDT (Hamblin et al., 1996; Nyman and Hynninen, 2004). For white or fluorescent lamps, it is very important to minimize ultra-violet emissions to avoid mutagenesis, as well as infrared, to minimize the risk of heating host tissues (Donnelly et al., 2008). In order to reduce damage to normal tissues, the light dose should not be higher than 200 mW/cm^2^ (Nyman and Hynninen, 2004; Donnelly et al., 2008). In addition, the light source should be chosen according to the targeted tissue, because the dose is dependent on the thickness of the tissue. For example, red light penetrates ∼3.0 nm whereas blue light penetrates ∼1.5 nm (Donnelly et al., 2008; Garland et al., 2009).

## Mechanism of Action

**FIGURE 1:**
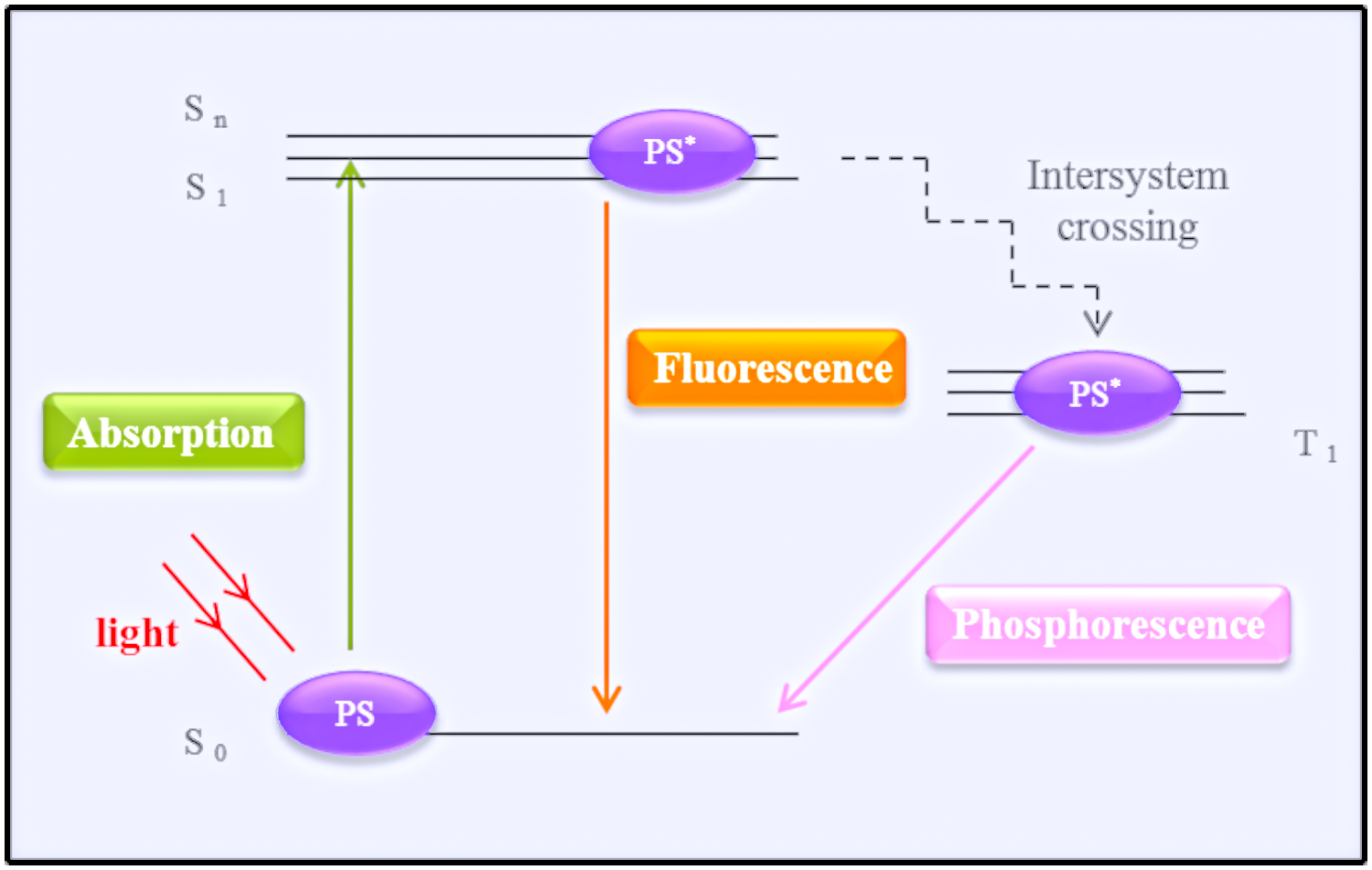
**Simplified schematic representation of a Jablonski diagram.** The photosensitizer (PS) at the ground state (S_0_) transitions after irradiation by a light source to its first single activate state (S_1_). To return to its ground state, the PS emits energy by fluorescence or phosphorescence (after reaching the triplet state – T_1_).

Photodynamic therapy typically induces the production of ROS and RNS (Hamblin and Hasan, 2004; Dai et al., 2012). The basic protocol of treatment involves PS administration followed by a wait time of varying duration to allow for the accumulation of the PS in the cells/tissue, after which the target tissue is irradiated with light source. The ground state of a PS is the singlet state (S_0_). Activation by irradiation results in the transit of electrons to a different orbital, exciting the PS to the form of an unstable molecule with a short half-life (first excited singlet-state, S_1_). In order to return to its stable ground state, the PS emits fluorescence or phosphorescence (by intersystem crossing; Nyman and Hynninen, 2004; Garland et al., 2009). Fluorescence emission does not alter the electron spin, phosphorescence changes in the spin rotation from an excited singlet-state to an excited triplet state, which has a long half-life (Hamblin and Hasan, 2004; Garland et al., 2009). The excited triplet state is the main mediator of the photodynamic reactions. The photophysical process is illustrated in **Figure 1**, using the energy levels, or Jablonski, diagram.

**FIGURE 2:**
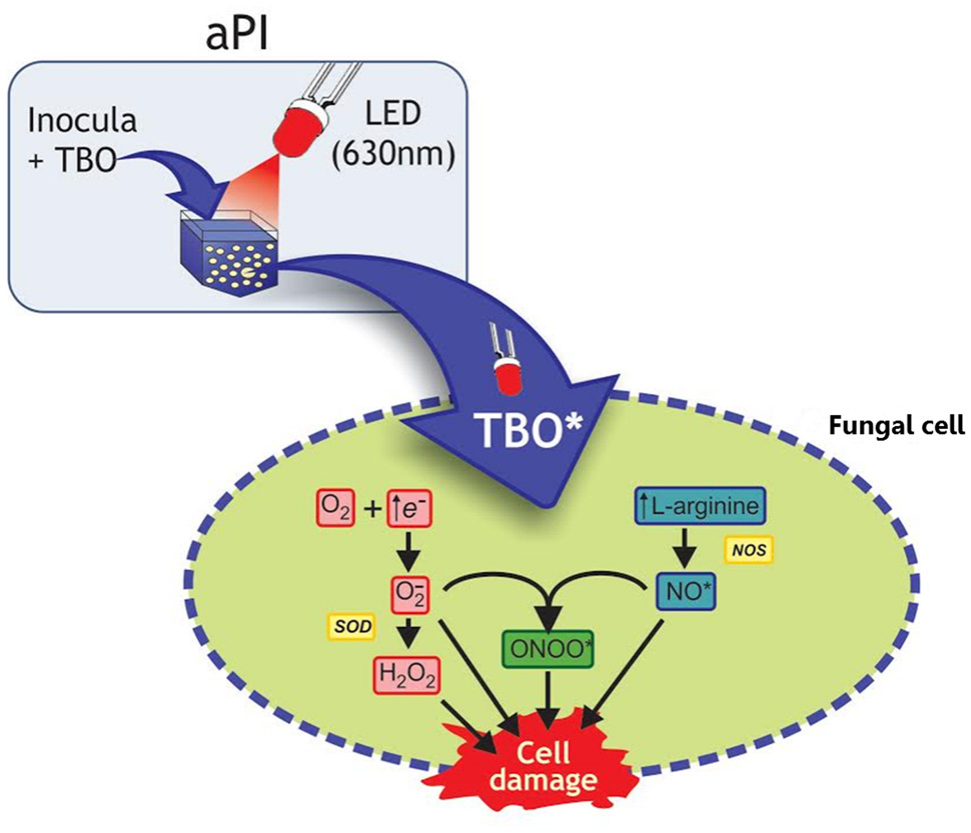
**Schematic illustration of the aPI (TBO + LED 630 nm) effects on fungal cell.** In this illustration with *Trichophyton rubrum* conidia, toluidine blue O (TBO) and LED light were used as example according to present the mechanism described by Baltazar Lde et al. (2013). Activation of TBO (both intra and extracellular) increases l-arginine levels, the substrate of oxide nitric synthase (NOS), which results in increasing NO^∙^ levels. The increased availability of free electrons increases H_2_O_2_ production. Moreover, NO^∙^ can react with O_2_^-^, generating ONOO^∙^. In eukaryotic cells generation of NO^∙^ occurs by oxidation of l-arginine. All these toxic radicals can react with the cell membrane and cytosolic components, leading to cell damage.

Two types of photodynamic reactions can occur, type 1 and type 2. In the type 1 reaction, the PS triplet directly transfers an electron or hydrogen to a biomolecule, producing reactive intermediates such as anion superoxide (O_2_^-^), hydrogen peroxide (H_2_O_2_), hydroxyl radials (OH^-^), nitric oxide (NO⋅), and peroxide nitrite (ONOO⋅; Hamblin and Hasan, 2004; Baltazar Lde et al., 2013; **Figure 2**). In the type 2 reaction, the PS transfers energy to molecular oxygen yielding the production of singlet oxygen (^1^O_2_), which is an extremely powerful oxidant with a very short life time, but it can react with several biomolecules, such as lipids and proteins (Hamblin and Hasan, 2004).

An important aspect of the generation of oxidative and nitrosative stresses by this process for antimicrobial applications is that the diverse cellular targets of these radicals reduces the probability of the selection of resistant strains, which is the main problem faced by the current antifungal therapies (Calzavara-Pinton et al., 2005, 2012). The radicals generated by PDT have extremely short half-lives and they react only in their sites of formation, which reduces their toxicity to adjacent normal tissues (Nyman and Hynninen, 2004).

The generated radicals alter the structure of the fungal cell wall and membrane, which provides the further translocation of the PS into the cell. Subsequently, these ROS and RNS produced outside and within the fungal cell cause an imbalance in cellular homeostasis, including damaging cytoplasmic organelles and nucleic acids, resulting in cell death by apoptosis, necrosis, or autophagy (Mroz et al., 2011). Interestingly, treatment using high doses of light and high concentrations of PS leads to cell death by necrosis, while treatment with low doses tends to induce cell death by apoptosis (Lennon et al., 1991; Noodt et al., 1996; Mroz et al., 2011). Depending on the amount of ROS produced and degree damage, death by autophagy can also occur (Mroz et al., 2011).

## *In vitro* Studies of Photodynamic Inhibition Targeting Fungal Cells

The *in vitro* effect of aPI against fungal cells has been demonstrated using different treatment regimes (**Table 1**). Takahashi et al. (2014) recently reported that *Malassezia furfur* is effectively killed using TONS504, a cationic PS, and 670 nm LED. The cidal effect on *M. furfur* is dose dependent and a reduction >80% was achieved using 100 J/cm^2^ and 1 μg/mL of light and PS, respectively. Smijs and Schuitmaker (2003) reported that *T. rubrum* cells are killed (based on a cut off of two colonies) after treatment with porphyrins deuteroporphyrin monomethylester (DP mme) and 5,10,15-tris(4-methylpyridinium)-20-phenyl-[21H,23H]-porphine trichloride (Sylsens B) in concentrations of 3 μg/mL or higher in combination with white light (1080 kJ/cm^2^). The study also highlights the possibility of using Sylsens B as a PS to treat tinea infections using red light to more deeply penetrate the skin. Kamp et al. (2005) showed that aPI using ALA (10 mmol l^-1^) as PS and quartz-halogen lamp (dose of 10 J) reduces *T. rubrum* growth by about 50% compared to untreated control conditions. Treating *T. rubrum* cells using a phenothiazine PS, Baltazar Lde et al. (2013), determined that aPI with TBO at a concentration of 10 μg/mL and an LED dose 48 J/cm^2^ is cidal to this microbe. Notably, this work also provides a description of the mechanism of action of aPI, which involves the production of ROS, NO⋅, and ONOO⋅. In addition, Baltazar et al. (2015) recently showed that curcumin (curc) and curcumin-nanoparticle (curc-np) aPI, at optimal conditions of 10 μg/mL of PS with 10 J/cm^2^ of blue light (417 ± 5 nm), completely inhibited *T. rubrum* growth via induction of ROS and RNS, which was associated with fungal death by apoptosis. Delivery of curc by nanoparticle enhanced apoptosis due to increased NO⋅ production. Romagnoli et al. (1998) reported that the combination of the thiophene 5-(4-OH-1-butinyl)-2,2^′^-bithienyl (BBTOH) with concentration of 50 μg/mL with UVA light (320–400 nm) over 90 min results in a >50% reduction in the growth of several dermatophytes, including *T. rubrum, T. mentagrophytes*, *T. tonsurans*, *Microsporum cookei*, *M. gypseum*, and *Epidermophyton floccosum,* with *E. floccosum* having the greatest susceptibility to this regimen.

**Table 1:** *In vitro* studies using antimicrobial photodynamic inhibition (aPI).

Fungus species	aPI	Final outcome	Reference
*Malassezia furfur*	TONS504, a cationic PS, and 670-nm LED	>80% reduction	Takahashi et al. (2014)
*Trichophyton rubrum*	3 μg/mL or higher of each deuteroporphyrin monomethylester (DP mme) or Sylsens B in and irradiation with white light (1080 kJ/cm^2^)	Eradication	Smijs and Schuitmaker (2003)
*T. rubrum*	ALA (10 m mol l^-1^) and quartz-halogen lamp (dose of 10 J/cm^2^)	50% reduction	Kamp et al. (2005)
*T. rubrum*	TBO concentration of 10 μg/mL and LED (dose 48 J/cm^2^)	Complete inhibition	Baltazar Lde et al. (2013)
*T. rubrum*	curc and curc-np aPI, at of 10 μg/mL of PS with 10 J/cm^2^ of blue light (417 ± 5 nm)	Complete inhibition	Baltazar et al. (2015)
*T. rubrum, T. mentagrophytes*, *T. tonsurans*, *Microsporum cookei*, *M. gypseum*, and *Epidermophyton floccosum*	Combination of BBTOH with UVA light	>50% reduction	Romagnoli et al. (1998)
*Candida albicans*	MB (concentrations of 0.027–0.27 mM) and laser (683 nm, 28 J/cm^2^) Germ tube formation: MB (concentrations 0.013 and 0.134 mM) with the same light dose	40% or more reduction depending on the PS concentration and >75% reduction in germ tube formation	Munin et al. (2007)
*C. albicans*	MB (0.05 mg/mL) and laser (684 nm, dose of 28 J/cm^2^).	50% reduction	Giroldo et al. (2009)
*C. albicans*	MB (0.05 mg/mL) and TBO (0.1 mg/mL) and two different LED lights (dose of 28 J/cm^2^) with wavelengths of 684 nm and 660 nm, respectively	80–90% reduction	Carvalho et al. (2009)
*C. albicans*, *C. tropicalis,* and *C. parapsilosis*	TBO (25 μM) and LED (dose of 180 J/cm^2^)	Inhibited *in vitro* growth and adhesion to buccal epithelial cells	Soares et al. (2009)
*C. albicans* wild-type and mutant	–	Mutant cells had reduced accumulation of the PS in the cytoplasm and reduced aPI killing	Prates et al. (2011)
*C. albicans*	Planktonic cells: curcumin (20 μM) and dose of blue LED (440–460 nm) of 5.28 J/cm^2^. Biofilm: LED dose of 5.28 J/cm^2^ and cumcumin concentration of 40 μM and time of pre-incubation of 5 and 20 min.	Eradication of planktonic cells and 68 and 87% reduction, depending on the pre-incubation time.	Dovigo et al. (2011)
*Cryptococcus neoformans*	Polycationic conjugate of polyethyleneimine and photosensitizer (PS) chlorin (e6; concentration of 10 μM) and LED 665 nm.	Cells were susceptible to photodynamic treatment	Fuchs et al. (2007)
*C. gattii*	TBO (25 μM) and LED (dose of 54 J/cm^2^).	Reduction of growth	Soares et al. (2011)
*C. neoformans* melanized cells	4.5 μM of CIAIPc/NE and light 675 nm (dose of 10 J/cm^2^).	Melanized cells were reduced up to 6 Logs	Rodrigues et al. (2012)
*C. neoformans*	MB, rose Bengal, EtNBSe, cationic fullerene, and conjugate between poly-l-lysine and chlorin (e6), each irradiated with appropriate light source	Cell wall, laccase, and melanin protected the cells	Prates et al. (2013)
*Sporothrix schenckii*	MB, NMB, or DMMB and LED (639.8 ± 10 nm) light dose of 37 J/cm^2^	6 Log_10_ fungicidal effect	Gilaberte et al. (2014)
*Fonsecaea pedrosoi* and *C. carrionii*.	MB (32 μg/mL) combined with LED (200 mW/cm^2^)	Cidality	Lyon et al. (2013)
*F. monophora*	ALA-PDT and LED (635 nm, 10 J)	Reduced CFUs and reduced colony diameter.	Hu et al. (2015)

In addition to the dermatophytes, aPI is effective against several yeast species. *Candida albicans* is a common microorganism used as model to study aPI. Munin et al. (2007) reported that aPI using MB (concentrations of 0.027–0.27 mM) and laser (683 nm, 28 J/cm^2^) reduced the growth of *C. albicans* in 40% or more, depending on the PS concentration. In addition, germ tube formation (the transition from a yeast cell to the hyphal form) was reduced by more than 75% using MB in the concentrations 0.013 and 0.134 mM with the same light dose. This work was the first to show the ability of aPI to inhibit the transition from yeast to hyphae cells, a step essential to the virulence of this species, suggesting that aPI could decrease the ability of *C. albicans* cells to cause disease. Similarly, Giroldo et al. (2009) found that MB (0.05 mg/mL) and laser (684 nm, dose of 28 J/cm^2^) reduced the viability of *C. albicans* by 50%. The results were associated with the permeabilization of the cells by MB, which damaged the plasma membrane. In addition, Carvalho et al. (2009) reported that aPI using MB (0.05 mg/mL) and TBO (0.1 mg/mL) and two different LED lights (dose of 28 J/cm^2^) with wavelengths of 684 and 660 nm, respectively, effectively decreased fungal viability by 80–90%. Notably, the phototoxic effect of MB was calcium dependent, a fact not observed with TBO, suggesting that they have different mechanisms of action against *C. albicans*. For MB, toxicity was related to alterations in plasma membrane calcium channels and the generation of ROS (Carvalho et al., 2009). Using TBO (25 μM) and LED (dose of 180 J/cm^2^), Soares et al. (2009) found that aPI reduced cell growth, reducing the median to Log_10_ 3.41 and adhesion ∼55% of different clinical isolates of *Candida* (*C. albicans*, *C. tropicalis,* and *C. parapsilosis*) to buccal epithelial cells. The study also reported that isolates that were resistant to fluconazole were susceptible to aPI.

Antimicrobial photodynamic inhibition efficacy may be impacted by cellular resistance strategies. Prates et al. (2011) reported that a *C. albicans* mutant overexpressing an ATP-binding cassette (ABC), a multidrug eﬄux system (MES), were not significantly damaged by MB-aPI due to the reduced accumulation of the PS in the cytoplasm. However, they showed that the combination of aPI with verapamil (an ABC inhibitor) increased MB uptake and enhanced the killing of *C. albicans*. Using curcumin (concentration of 20 μM) as PS, Dovigo et al. (2011) showed complete inactivation of planktonic *C. albicans* after irradiation by blue LED (440–460 nm) of 5.28 J/cm^2^. The study suggested that curcumin could either bind to or be taken up by the planktonic yeast cells. However, the efficacy of this approach was lower in the setting of biofilm growth as the same light dose. Nevertheless, higher concentration of curcumin (40 μM) with different pre-incubation times (of 5 or 20 min) reduced viability to 68 and 87%, respectively. Additionally, the conditions used for planktonic *C. albicans* cells were also toxic to macrophages, which limits the systemic clinical application of this approach.

Antimicrobial photodynamic inhibition is also effective against *Cryptococcus* sp. Fuchs et al. (2007) showed that *Cryptococcus neoformans* was susceptible (killing at a Log_10_ of 2) to a polycationic conjugate of polyethyleneimine and the PS chlorin (e6; concentration of 10 μM) and LED 665 nm. The importance of cell wall integrity in the outcome of aPI was demonstrated in this work using a *C. neoformans* mutant *rom2* (with alterations in cell wall integrity) in which they found that the *rom2* mutant accumulated higher amounts of the PS inside the cell cytoplasm compared to wild-type. Using TBO (25 μM) and LED (dose of 54 J/cm^2^), Soares et al. (2011) reported the efficacy of aPI in a set of *C. gattii* isolates with different susceptibility profiles to antifungal drugs, suggesting that aPI could be an alternative tool to inhibit *C. gattii* growth. The pattern of reduction was variable among the strains which showed reduction of viability in the range of 1.78 Log_10_ to 6.45 Log_10_. The study also reported that aPI induced massive production of ROS/ONOO⋅, which was correlated to its killing effect; however, higher catalase and peroxidase activities were related with lower susceptibility to aPI.

Supporting the role of cell wall integrity in modifying the efficacy of aPI, Rodrigues et al. (2012) found that melanized *C. neoformans* cells were killed (up to 6 Logs) by aPI with 4.5 μM of CIAIPc in nanoemulsion (CIAIPc/NE) and light 675 nm (dose of 10 J/cm^2^). The study also showed that using lower CIAIPc/NE concentration (0.045 μM) and lower light dose (5 J/cm^2^) melanized cells had slightly reduced susceptibility compared non-melanized cells. Similar results were found by Prates et al. (2013) using five different PSs [MB, rose Bengal, selenium derivative of a Nile blue (EtNBSe), *tris*-cationic fullerene (BB6), and conjugate between poly-l-lysine and chlorin (e6)] and an appropriate light source. The presence of cell wall, laccase (the enzyme responsible for melanization of *C. neoformans*) and melanin protected the cells from the harmful effects related with aPI, but cidality could nevertheless be achieved with certain combinations of PS and light dose.

Similar to the work with dermatophytes, aPI has been successfully employed against agents of subcutaneous mycosis such as *Sporothrix schenckii*, *Fonsecaea pedrosoi,* and *Cladosporium carrionii*. Gilaberte et al. (2014) described the success of aPI against *S. schenckii*, obtaining a 6 Log_10_ fungicidal effect using different phenothiazinium PSs [MB, new methylene blue (NMB), or 1,9-dimethylmethylene blue (DMMB)] and LED (639.8 ± 10 nm) light dose of 37 J/cm^2^. Lyon et al. (2013) reported that MB (32 μg/mL) combined with LED (200 mW/cm^2^) was effective in killing *F. pedrosoi* and *C. carrionii*. These two pathogens are etiological agents of chromoblastomycosis, which is a disease that is severely resistant to standard antifungal treatment. Hence, these results indicate that aPI could be a promising approach to chromoblastomycosis. Hu et al. (2015) reported that ALA-PDT and LED (635 nm, 10 J) also reduced the viability of *F. monophora*.

## *In vivo* Studies

The *in vitro* efficacy of aPI against different fungal pathogens has been confirmed *in vivo* using various animal models (**Table 2**). In a murine cutaneous *C. albicans* infection model, Dai et al. (2011) evaluated aPDT using NMB and red light (at 635 ± 15 nm or 660 ± 15 nm delivered at 78 J/cm^2^ for “prophylaxis” at 30 min or 120 J/cm^2^ at 24 h for treatment post-infection). A luciferase-expressing strain of *C. albicans* was used to allow real-time monitoring through bioluminescence imaging. aPDT initiated either at 30 min or at 24 h post-infection significantly reduced the *C. albicans* burden 95.4 and 97.4%, respectively, compared to controls. Teichert et al. (2002) evaluated the efficacy of MB-mediated PDT to treat oral candidiasis in an immunosuppressed murine model, in an attempt to mimic thrush in patients. Mice with severe immunodeficiency disease were inoculated orally with *C. albicans* by swab three times a week for a 4-week period. Before treatment, mice were cultured for baseline fungal growth and received a topical oral cavity administration of 0.05 mL MB solution at different concentrations (250, 275, 300, 350, 400, 450, or 500 μg/mL). After of 10 min of MB solution treatment, mice were irradiated with light at 664 nm using a diode laser light with a cylindrical diffuser. MB aPDT had a dose-dependent effect as concentrations from 250 to 400 μg/mL reduced fungal growth but did not eliminate *C. albicans* while concentrations of 450 and 500 μg/mL totally eradicated the fungus from the oral cavity. Junqueira et al. (2009) evaluated the effects of aPDT on buccal candidiasis using a rat model. After inducing candidiasis on the dorsal aspect of rat tongues, aPDT was achieved using a laser and MB, and *Candida* colonization, epithelial alterations, and chronic inflammation were analyzed using histology. The effect was more visible on day 5 after treatment; at day 5 treated rats had fewer epithelial alterations (pathological score 1.00 treated and 1.50 in control group) and less chronic inflammation (pathological score 1.00 treated and 1.50 in control) than control animals. Mima et al. (2010) conducted an *in vivo* oral candidiasis study in immunosuppressed mice to evaluate the efficacy of aPDT of oral candidiasis using Photogem, a hematoporphyrin derivative, at 400, 500, or 1000 mg/L which was followed 30 min later by illumination with LED light (305 J/cm^2^) at 455 or 630 nm. aPDT resulted in 1.05, 1.59, and 1.40 log_10_ reductions, respectively, in tongue *C. albicans* colony counts; however, there was no difference in fungal burden between the concentrations of Photogem and LED light wavelengths used. Notably, histological evaluation of the tongue revealed that aPDT did not cause any significant adverse effects to the local mucosa. A murine oral candidiasis model was also utilized to explore the efficacy of curcumin as a PS (Dovigo et al., 2013). Five days after *C. albicans* infection, mice received topical curcumin (20, 40, and 80 μM) and illumination with LED light at 455 nm. This treatment significantly reduced the *C. albicans* viability in a dose dependent manner with 80 μM of curcumin associated with light leading to the highest reduction, 4 logs, in colony counts.

**Table 2:** Pre-clinical studies.

Disease	Fungus species	aPDT	Synergist	Final outcome	Reference
Murine model of cutaneous candidiasis	*C. albicans*	NMB-red light		Control the fungal burden in the skin	Dai et al. (2011)
Murine model of oral candidiasis	*C. albicans*	MB- diode laser light	–	Eradicated the fungus from the oral cavity	Teichert et al. (2002)
Rat model of buccal candidiasis	*C. albicans*	MB-leser		Reduction in chronic inflammation	Junqueira et al. (2009)
Murine model of oral candidiasis	*C. albicans*	Photogem^®^ and LED		Reduced fungal burden	Mima et al. (2010)
Murine model of oral candidiasis	*C. albicans*	Curcumin-LED	–	Reduced fungal viability and fungal burden	Dovigo et al. (2013)
Murine model of ear pinna infection	*C. albicans*	TMP-1363 and Irradiation	–	Controled the fungal burden in the pinna	Mitra et al. (2011)
Murine model of vaginitis	*C. albicans*	MB and red laser	–	Reduced fungal growth and decreased inflammatory cells	Machado-de-Sena et al. (2014)
Murine model of Dermatophytosis	*T. rubrum*	TBO-LED	CPX, 0.65 mg/mice	Reduced fungal burden and decreased skin damage	Baltazar et al. (2014)
Candiditis on* Galleria mellonella*	*C. albicans*	MB and red light	Fluconazole	Reduced fungal burden and prolonged survival	Chibebe Junior et al. (2013)

Mitra et al. (2011) investigated the efficacy of aPDT for the treatment of *C. albicans* ear pinna infection using a mouse model. They selected TMP-1363 as the PS after showing its efficacy for killing *C. albicans in vitro*. The intradermal space of the ear pinna was inoculated with *C. albicans*. After 2 days, 0.3 mg/mL TMP-1363 was administered topically and the ears were irradiated at 514 nm using a fluence of 90 J/cm^2^ delivered at an irradiance of 50 mW/cm^2^. aPDT with TMP-1363 resulted in a 50-fold reduction of *C. albicans* CFU/ear compared to untreated controls, and the infected ears subjected to aPDT completely healed over time without any residual damage to the pinna. Machado-de-Sena et al. (2014) evaluated the efficacy of aPDT for treatment of *C. albicans* vaginal infection using MB and red light. Mice were inoculated intravaginally with *C. albicans*, and then were treated with aPDT 5 days later using MB and red laser. This approach significantly reduced *C. albicans* growth 1.66 log CFU/mL and percentages of inflammatory area were significantly reduced with just two sessions of aPDT.

Recently, we reported the *in vivo* application of aPDT against *T. rubrum* (Baltazar et al., 2014). C57BL/6 mice were cutaneously infected with *T. rubrum* and treated with aPDT for 7 days every 24 h using a TBO 0.2% gel formulation and an LED 630 nm dose of 42 J/cm^2^. aPDT was compared to treatment with the antifungal cyclopiroxolamine (CPX, 0.65 mg/mice) administered topically every 48 h for 7 days. aPDT was 64% more efficient than CPX in reducing the fungal burden, and both treatments reduced the damage caused by the fungus in the skin. aPDT also reduced myeloperoxidase (MPO) levels, but not the activity of *N*-acetylglucosaminidase (NAG), suggesting that there was a reduction in neutrophils but not macrophages in the affected tissues. Furthermore, the study associated the effective production of ROS with aPDT efficacy.

Antimicrobial photodynamic inhibition of *C. albicans* was also studied using the non-vertebrate host *Galleria mellonella*, the wax moth (Chibebe Junior et al., 2013). aPDT MB with red light significantly reduced the fungal burden and prolonged the survival of *C. albicans* infected *G. mellonella* larvae compared to controls. A fluconazole-resistant *C. albicans* strain was also used to test the combination of aPDT and fluconazole, and this combined approach significantly prolonged the survival of the larvae compared to each individual treatment alone.

## PDT for Human Fungal Infections

The increased incidence of drug resistant pathogens has led investigators to explore innovative approaches to infectious diseases in clinical studies, including using aPI to combat fungal infections. In this section, we highlight the human studies for the treatment of fungal infection using aPDT (**Table 3**).

**Table 3:** Clinical trials using aPDT as treatment.

Disease	Fungus species	aPDT	Additional treatment	Final outcome	Reference
Pityriasis versicolor	*Malassezia* species	ALA-PDT	–	Complete clearance	Kim and Kim (2007)
Onychomycosis	*T. rubrum*	ALA-PDT	Treatment with 40% urea ointment for 12 h prior to aPDT	Clinical and microbiological cures	Piraccini et al. (2008)
Onychomycosis	*–*	ALA and red light	–	Significant improvement after treatments	Sotiriou et al. (2010)
*Malassezia* infection	*M. folliculitis*	MAL-PDT		Control of the tissue fungal burden	Lee et al. (2010)
Onychomycosis	*F. oxysporum* and *A. terreus*	MAL 16% and LED	Treatment with 40% urea ointment for 12 h prior to aPDT	Clinical and microbiological cures	Gilaberte et al. (2011)
Denture stomatitis (DS)	*Candida* species	Photogem^®^ and LED	–	Mycological cultures	Mima et al. (2012)
Sporotrichosis	*S. schenckii* complex	MB-PDT	Low dose itraconazole	Complete microbiological and clinical response	Gilaberte et al. (2014)
Chromoblastomycosis	*F. pedrosoi* and *C. carrionii*	MB and LED light		Control of the tissue fungal burdens	Lyon et al. (2011)

Kim and Kim (2007) using ALA-PDT (two sessions) and red light (70–100 J/cm^2^) showed the efficacy of this approach for treating pityriasis versicolor. The study reported that there were no hyphae or spores found in the infected area at 10 days after treatment. Piraccini et al. (2008) described the success of onychomycosis (caused by *T. rubrum*) treatment using aPDT in a patient with had failed to respond to the treatments with conventional topical antifungal drugs. Before each session (three sessions at 15 day intervals between treatments), the patient’s nail was first coated with a 40% urea ointment that was then kept under occlusion for 7 days to soften the plate and then the diseased nail was subjected to aPDT using ALA (160 mg/g) and red light (630 nm, 37 J/cm^2^). After the three aPDT sessions during a period of 45 days, the patient was evaluated every 3 months for 24 months. Cultures were positive at the third aPDT session, but became negative 3 months after the last treatment. At the 12th month visit, cultures were still negative and the toenails were considered clinically cured and disease had not recurred at the 24th month evaluation. In another clinical trial of 30 patients with onychomycosis, patients that received aPDT therapy combining ALA and red light (570–670 nm, dose of 40 J/cm^2^) had a 43% cure rate at 12 months after the treatment and 37% remained disease free at 18 months (Sotiriou et al., 2010). Lee et al. (2010) reported that four of six patients with recalcitrant *Malassezia* folliculitis demonstrated significant improvement after treatment with methyl 5-ALA (MAL)-PDT and red LED light (630 nm, light dose of 37 J/cm^2^).

Gilaberte et al. (2011), reported treatment of two patients with fingernail onychomycosis unresponsive to standard antifungals with disease caused by the *Fusarium oxysporum* or *Aspergillus terreus*. In both cases, the nail plate was first softened with 40% urea ointment under occlusion for 12 h. aPDT was performed using MAL 16% cream and illumination using a 635-nm LED (dose of 37 J/cm^2^). A single treatment clinically improved the nail appearance and cultures were thereafter negative. Two additional treatments were administered and both patients remained disease free during subsequent follow up evaluations.

Mima et al. (2012) compared aPDT with topical antifungal cream for the treatment of denture stomatitis (DS) caused by *Candida* species. Patients were randomly assigned (*n* = 20 each) to receive either nystatin (NYT) or aPDT. In the NYT group, patients received topical treatment with nystatin (100 000 IU) four times daily during 15 days. The aPDT group each had 500 mg/L of Photogem^®^ applied to their dentures and palates. After 30 min of incubation, the Photogem coated surfaces were illuminated with LED light (455 nm, doses of 37.5 and 122 J/cm^2^, respectively) three times a week for 15 days. Mycological cultures were taken from dentures at baseline (day 0), at the end of the treatment (day 15) and at the follow-up time intervals (days 30, 60, and 90). Both treatments significantly reduced the fungal burden at the end of the treatments and on day 30 of the follow-up period; however, there were no significant differences between the two treatment modalities (53 vs. 45% for NYT and PDT, respectively). The study highlighted that fewer sessions of aPDT were necessary to achieve the same result that NYT achieved, albeit the NYT approach did not require clinic visits.

Gilaberte et al. (2014) used aPDT in a patient with recalcitrant cutaneous sporotrichosis. They used intralesional applications of 1% MB and LED light at 635 nm to administer 37 J/cm^2^ to each lesion in combination with low doses of itraconazole (100 mg/day). The aPDT was performed three times every other week and this approach resulted in a complete clinical and microbiological cure (Gilaberte et al., 2014). In a clinical trial, Lyon et al. (2011) treated 10 patients with chromoblastomycosis with a combination of MB and red LED (660 nm, dose of 28 J/cm^2^). The patients underwent six treatment sessions (every week) and, although the treatment did not result in complete healing of the lesions, aPDT resulted in clear reductions of the volume and cicatrization of 80–90% of the lesions.

## Perspectives and Conclusion

The significant burden of dermatophytoses and the worldwide increase in fungal strains resistant to the current antifungals (Cowen, 2008) increases the urgency for the development of new therapeutic strategies, such as aPDT. In this review we described *in vitro* and *in vivo* studies as well as the few, small human experiences and trials that support the development of the aPDT either as adjuvant or as a primary therapeutic approach against cutaneous mycoses.

The *in vitro* mechanism of action described thus far demonstrate that aPI induces the generation of ROS and RNS, which effectively damage a range of fungal cellular structures and induce cell death. There are no reports of mutagenic or genotoxic effects to the fungal or human cells. However, there is an ongoing need for deeper study of the mechanisms of aPDT to facilitate the expanded clinical use of this promising therapeutic approach. Although the majority of aPDT studies have focused on *Candida* biofilms, it is important to also investigate the application of this approach using other major fungal pathogens, including *Cryptococcus* species or *Coccidioides* species where biofilm formation contributes to disease severity in the central nervous system (Sardi Jde et al., 2014). The incorporation of PSs into liposomes, micelles, or nanoparticles is a promising approach to reduce the PS self-aggregation and to enhance the targeted delivery of the PS. The development of these vehicles is particularly important for the potential expansion of aPDT for the treatment of deep fungal infections using fiber optic lasers, applied endoscopically, or interstitially.

## Conflict of Interest Statement

The authors declare that the research was conducted in the absence of any commercial or financial relationships that could be construed as a potential conflict of interest.

## References

[B1] Baltazar L. M. Krausz A. E. Souza A. C. O. Adler B. L. Landriscina A. Musaev T. (2015). Trichophyton rubrum is inhibited by free and nanoparticle encapsulated curcumin by induction of nitrosative stress after photodynamic activation. *PLoS ONE* (in press). 10.1371/journal.pone.0120179 PMC4372525 25803281

[B2] Baltazar Lde M. Soares B. M. Carneiro H. C. Avila T. V. Gouveia L. F. Souza D. G. (2013). Photodynamic inhibition of *Trichophyton rubrum*: in vitro activity and the role of oxidative and nitrosative bursts in fungal death. *J. Antimicrob. Chemother.* 68 354–361 10.1093/jac/dks414 23134678

[B3] Baltazar L. M. Werneck S. M. Carneiro H. C. Gouveia L. F. De Paula T. P. Byrro R. M. (2014). photodynamic therapy efficiently controls dermatophytosis caused by *Trichophyton rubrum* in a murine model. *Br. J. Dermatol.* 10.1111/bjd.13494 [Epub ahead of print]. 25350570

[B4] Brown G. D. Denning D. W. Gow N. A. Levitz S. M. Netea M. G. White T. C. (2012). Hidden killers: human fungal infections. *Sci. Transl. Med*. 4 165rv13 10.1126/scitranslmed.3004404 23253612

[B5] Calin M. A. Parasca S. V. (2009). Light sources for photodynamic inactivation of bacteria. *Lasers Med. Sci*. 24 453–460 10.1007/s10103-008-0588-5 18622686

[B6] Calzavara-Pinton P. Rossi M. T. Sala R. Venturini M. (2012). Photodynamic antifungal chemotherapy. *Photochem. Photobiol.* 88 512–522 10.1111/j.1751-1097.2012.01107.x 22313493

[B7] Calzavara-Pinton P. G. Venturini M. Sala R. (2005). A comprehensive overview of photodynamic therapy in the treatment of superficial fungal infections of the skin. *J. Photochem. Photobiol. B Biol.* 78 1–6 10.1016/j.jphotobiol.2004.06.006. 15629243

[B8] Carvalho G. G. Felipe M. P. Costa M. S. (2009). The photodynamic effect of methylene blue and toluidine blue on *Candida albicans* is dependent on medium conditions. *J. Microbiol.* 47 619–623 10.1007/s12275-009-0059-0 19851735

[B9] Chibebe Junior J. Sabino C. P. Tan X. Junqueira J. C. Wang Y. Fuchs B. B. (2013). Selective photoinactivation of *Candida albicans* in the non-vertebrate host infection model *Galleria mellonella*. *BMC Microbiol.* 13:217 10.1186/1471-2180-13-217 PMC3849975 24083556

[B10] Cormick M. P. Alvarez M. G. Rovera M. Durantini E. N. (2009). Photodynamic inactivation of *Candida albicans* sensitized by tri- and tetra-cationic porphyrin derivatives. *Eur. J. Med. Chem*. 44 1592–1599 10.1016/j.ejmech.2008.07.026 18762356

[B11] Cowen L. E. (2008). The evolution of fungal drug resistance: modulating the trajectory from genotype to phenotype. *Nat. Rev. Microbiol.* 6 187–198 10.1038/nrmicro1835 18246082

[B12] Craig R. A. Mccoy C. P. Gorman S. P. Jones D. S. (2014). Photosensitisers – the progression from photodynamic therapy to anti-infective surfaces. *Expert. Opin. Drug. Deliv.* 12 85–101 10.1517/17425247.2015.962512 25247277

[B13] Dai T. Bil De Arce V. J. Tegos G. P. Hamblin M. R. (2011). Blue dye and red light, a dynamic combination for prophylaxis and treatment of cutaneous *Candida albicans* infections in mice. *Antimicrob. Agents Chemother.* 55 5710–5717 10.1128/AAC.05404-11 21930868 PMC3232820

[B14] Dai T. Fuchs B. B. Coleman J. J. Prates R. A. Astrakas C. St Denis T. G. (2012). Concepts and principles of photodynamic therapy as an alternative antifungal discovery platform. *Front. Microbiol.* 3:120 10.3389/fmicb.2012.00120 PMC3322354 22514547

[B15] Donnelly R. F. Mccarron P. A. Tunney M. M. (2008). Antifungal photodynamic therapy. *Microbiol. Res.* 163 1–12 10.1016/j.micres.2007.08.001 18037279

[B16] Dougherty T. J. Kaufman J. E. Goldfarb A. Weishaupt K. R. Boyle D. Mittleman A. (1978). Photoradiation therapy for the treatment of malignant tumors. *Cancer Res.* 38 2628–2635. 667856

[B17] Dovigo L. N. Carmello J. C. De Souza Costa C. A. Vergani C. E. Brunetti I. L. Bagnato V. S. (2013). Curcumin-mediated photodynamic inactivation of *Candida albicans* in a murine model of oral candidiasis. *Med. Mycol*. 51 243–251 10.3109/13693786.2012.714081 22934533

[B18] Dovigo L. N. Pavarina A. C. Ribeiro A. P. Brunetti I. L. Costa C. A. Jacomassi D. P. (2011). Investigation of the photodynamic effects of curcumin against *Candida albicans*. *Photochem. Photobiol.* 87 895–903 10.1111/j.1751-1097.2011.00937.x 21517888

[B19] Drake L. A. Dinehart S. M. Farmer E. R. Goltz R. W. Graham G. F. Hordinsky M. K. (1996). Guidelines of care for superficial mycotic infections of the skin: tinea capitis and tinea barbae. Guidelines/Outcomes Committee. American Academy of Dermatology. *J. Am. Acad. Dermatol.* 34 290–294 10.1016/S0190-9622(96)80137-X 8642096

[B20] El-Gohary M. Van Zuuren E. J. Fedorowicz Z. Burgess H. Doney L. Stuart B. (2014). Topical antifungal treatments for tinea cruris and tinea corporis. *Cochrane Database Syst. Rev.* 8 CD009992 10.1002/14651858.CD009992.pub2 PMC11198340 25090020

[B21] Fuchs B. B. Tegos G. P. Hamblin M. R. Mylonakis E. (2007). Susceptibility of *Cryptococcus neoformans* to photodynamic inactivation is associated with cell wall integrity. *Antimicrob. Agents Chemother.* 51 2929–2936 10.1128/AAC.00121-07 17548495 PMC1932496

[B22] Gaitanis G. Velegraki A. Mayser P. Bassukas I. D. (2013). Skin diseases associated with *Malassezia* yeasts: facts and controversies. *Clin. Dermatol.* 31 455–463 10.1016/j.clindermatol.2013.01.012 23806162

[B23] Garland M. J. Cassidy C. M. Woolfson D. Donnelly R. F. (2009). Designing photosensitizers for photodynamic therapy: strategies, challenges and promising developments. *Future Med*.*Chem*. 1 667–691 10.4155/fmc.09.55 21426032

[B24] Gilaberte Y. Aspiroz C. Alejandre M. C. Andres-Ciriano E. Fortuno B. Charlez L. (2014). Cutaneous sporotrichosis treated with photodynamic therapy: an in vitro and in vivo study. *Photomed. Laser Surg.* 32 54–57 10.1089/pho.2013.3590 24328608 PMC3887415

[B25] Gilaberte Y. Aspiroz C. Martes M. P. Alcalde V. Espinel-Ingroff A. Rezusta A. (2011). Treatment of refractory fingernail onychomycosis caused by nondermatophyte molds with methylaminolevulinate photodynamic therapy. *J. Am. Acad. Dermatol.* 65 669–671 10.1016/j.jaad.2010.06.008 21839332

[B26] Giroldo L. M. Felipe M. P. De Oliveira M. A. Munin E. Alves L. P. Costa M. S. (2009). Photodynamic antimicrobial chemotherapy (PACT) with methylene blue increases membrane permeability in *Candida albicans*. *Lasers Med. Sci*. 24 109–112 10.1007/s10103-007-0530-2 18157564

[B27] Gupta A. K. Cooper E. A. (2008). Update in antifungal therapy of dermatophytosis. *Mycopathologia* 166 353–367 10.1007/s11046-008-9109-0 18478357

[B28] Hamblin M. R. Hasan T. (2004). Photodynamic therapy: a new antimicrobial approach to infectious disease? *Photochem*.*Photobiol. Sci.* 3 436–450 10.1039/b311900a PMC3071049 15122361

[B29] Hamblin M. R. Miller J. L. Hasan T. (1996). Effect of charge on the interaction of site-specific photoimmunoconjugates with human ovarian cancer cells. *Cancer Res.* 56 5205–5210. 8912858

[B30] Harris F. Pierpoint L. (2012). Photodynamic therapy based on 5-aminolevulinic acid and its use as an antimicrobial agent. *Med. Res. Rev*. 32 1292–1327 10.1002/med.20251 21793017

[B31] Hausman W. (1911). Die sensibilisierende wirkung des hematoporphyrins. *Biochem. Z.* 30 276.

[B32] Hu Y. Huang X. Lu S. Hamblin M. R. Mylonakis E. Zhang J. (2015). Photodynamic therapy combined with terbinafine against chromoblastomycosis and the effect of PDT on *Fonsecaea monophora* in vitro. *Mycopathologia* 179 103–119 10.1007/s11046-014-9828-3 25366276 PMC4323679

[B33] Jesionek H. Tappeiner H. V. (1903). Therapeutische Versuche mit Fluoreszierenden Stoffen. *Muench. Med. Wochneshr*. 47 2024–2044.

[B34] Junqueira J. C. Martins Jda S. Faria R. L. Colombo C. E. Jorge A. O. (2009). Photodynamic therapy for the treatment of buccal candidiasis in rats. *Lasers Med. Sci*. 24 877–884 10.1007/s10103-009-0673-4 19408038

[B35] Kamp H. Tietz H. J. Lutz M. Piazena H. Sowyrda P. Lademann J. (2005). Antifungal effect of 5-aminolevulinic acid PDT in *Trichophyton rubrum*. *Mycoses* 48 101–107 10.1111/j.1439-0507.2004.01070.x 15743426

[B36] Kim Y. J. Kim Y. C. (2007). Successful treatment of pityriasis versicolor with 5-aminolevulinic acid photodynamic therapy. *Arch. Dermatol.* 143 1218–1220 10.1001/archderm.143.9.1218 17875898

[B37] Krausz A. Friedman A. J. (2014). News, views, & reviews: antimicrobial photodynamic therapy: applications beyond skin cancer. *J. Drugs Dermatol.* 13 624–626. 24936602

[B38] Lee J. W. Kim B. J. Kim M. N. (2010). Photodynamic therapy: new treatment for recalcitrant *Malassezia* folliculitis. *Lasers Surg. Med*. 42 192–196 10.1002/lsm.20857 20166153

[B39] Lennon S. V. Martin S. J. Cotter T. G. (1991). Dose-dependent induction of apoptosis in human tumour cell lines by widely diverging stimuli. *Cell Prolif.* 24 203–214 10.1111/j.1365-2184.1991.tb01150.x 2009322

[B40] Lipson R. L. Baldes E. J. (1960). The photodynamic properties of a particular hematoporphyrin derivative. *Arch. Dermatol.* 82 508–516 10.1001/archderm.1960.01580040026005 13762615

[B41] Lyon J. P. Moreira L. M. De Carvalho V. S. Dos Santos F. V. De Lima C. J. De Resende M. A. (2013). In vitro photodynamic therapy against *Fonsecaea pedrosoi* and *Cladophialophora carrionii*. *Mycoses* 56 157–161 10.1111/j.1439-0507.2012.02226.x 22816425

[B42] Lyon J. P. Pedroso e Silva Azevedo Cde M. Moreira L. M. De Lima C. J. De Resende M. A. (2011). Photodynamic antifungal therapy against chromoblastomycosis. *Mycopathologia* 172 293–297 10.1007/s11046-011-9434-6 21643843

[B43] Machado-de-Sena R. M. Correa L. Kato I. T. Prates R. A. Senna A. M. Santos C. C. (2014). Photodynamic therapy has antifungal effect and reduces inflammatory signals in *Candida albicans*-induced murine vaginitis. *Photodiagnosis Photodyn. Ther.* 11 275–282 10.1016/j.pdpdt.2014.03.013 24792453

[B44] Mantareva V. Angelov I. Kussovski V. Dimitrov R. Lapok L. Wohrle D. (2011). Photodynamic efficacy of water-soluble Si(IV) and Ge(IV) phthalocyanines towards *Candida albicans* planktonic and biofilm cultures. *Eur. J. Med. Chem*. 46 4430–4440 10.1016/j.ejmech.2011.07.015 21816518

[B45] Meyer-Betz F. (1913). Untersuchungen uber die Biologische (photodynamische) Wirkung des Hamatoporphyrins und anderer Derivate des Blut – und Galenfarbstoffs. *Dtsch. Arch. Klin. Med*. 112 475–503.

[B46] Mima E. G. Pavarina A. C. Dovigo L. N. Vergani C. E. Costa C. A. Kurachi C. (2010). Susceptibility of *Candida albicans* to photodynamic therapy in a murine model of oral candidosis. *Oral Surg. Oral Med. Oral Pathol. Oral Radiol. Endod*. 109 392–401 10.1016/j.tripleo.2009.10.006 20060338

[B47] Mima E. G. Vergani C. E. Machado A. L. Massucato E. M. Colombo A. L. Bagnato V. S. (2012). Comparison of photodynamic therapy versus conventional antifungal therapy for the treatment of denture stomatitis: a randomized clinical trial. *Clin. Microbiol. Infect.* 18 E380–E388 10.1111/j.1469-0691.2012.03933.x 22731617

[B48] Mitra S. Haidaris C. G. Snell S. B. Giesselman B. R. Hupcher S. M. Foster T. H. (2011). Effective photosensitization and selectivity in vivo of *Candida Albicans* by meso-tetra (*N*-methyl-4-pyridyl) porphine tetra tosylate. *Lasers Surg. Med*. 43 324–332 10.1002/lsm.21049 21500227 PMC3080247

[B49] Mroz P. Yaroslavsky A. Kharkwal G. B. Hamblin M. R. (2011). Cell death pathways in photodynamic therapy of cancer. *Cancers (Basel)* 3 2516–2539 10.3390/cancers3022516 23914299 PMC3729395

[B50] Mukherjee P. K. Leidich S. D. Isham N. Leitner I. Ryder N. S. Ghannoum M. A. (2003). Clinical *Trichophyton rubrum* strain exhibiting primary resistance to terbinafine. *Antimicrob. Agents Chemother.* 47 82–86 10.1128/AAC.47.1.82-86.2003 12499173 PMC148991

[B51] Munin E. Giroldo L. M. Alves L. P. Costa M. S. (2007). Study of germ tube formation by *Candida albicans* after photodynamic antimicrobial chemotherapy (PACT). *J. Photochem. Photobiol. B Biol.* 88 16–20 10.1016/j.jphotobiol.2007.04.011 17566757

[B52] Nenoff P. Grunewald S. Paasch U. (2014a). Laser therapy of onychomycosis. *J. Dtsch. Dermatol. Ges.* 12 33–38 10.1111/ddg.12251 24237592

[B53] Nenoff P. Kruger C. Ginter-Hanselmayer G. Tietz H. J. (2014b). Mycology – an update. Part 1: Dermatomycoses: causative agents, epidemiology and pathogenesis. *J. Dtsch. Dermatol. Ges*. 12 188–209; quiz 210 188–211; quiz 212. 10.1111/ddg.12245 24533779

[B54] Noodt B. B. Berg K. Stokke T. Peng Q. Nesland J. M. (1996). Apoptosis and necrosis induced with light and 5-aminolaevulinic acid-derived protoporphyrin IX. *Br. J. Cancer* 74 22–29 10.1038/bjc.1996.310 8679453 PMC2074599

[B55] Nyman E. S. Hynninen P. H. (2004). Research advances in the use of tetrapyrrolic photosensitizers for photodynamic therapy. *J. Photochem. Photobiol. B Biol.* 73 1–28 10.1016/j.jphotobiol.2003.10.002 14732247

[B56] Pathak M. A. Fitzpatrick T. B. (1992). The evolution of photochemotherapy with psoralens and UVA (PUVA): 2000 BC to 1992 AD. *J. Photochem. Photobiol. B Biol.* 14 3–22 10.1016/1011-1344(92)85080-E 1432383

[B57] Piraccini B. M. Rech G. Tosti A. (2008). Photodynamic therapy of onychomycosis caused by *Trichophyton rubrum*. *J. Am. Acad. Dermatol.* 59 S75–S76 10.1016/j.jaad.2008.06.015 19119130

[B58] Plaetzer K. Krammer B. Berlanda J. Berr F. Kiesslich T. (2009). Photophysics and photochemistry of photodynamic therapy: fundamental aspects. *Lasers Med. Sci*. 24 259–268 10.1007/s10103-008-0539-1 18247081

[B59] Policard A. (1924). Etudes sur les aspects offerts par des tumeurs experimentales examinees a la lumiere de Wood. *Cr. Soc. Biol.* 91 1423–1428.

[B60] Prates R. A. Fuchs B. B. Mizuno K. Naqvi Q. Kato I. T. Ribeiro M. S. (2013). Effect of virulence factors on the photodynamic inactivation of *Cryptococcus neoformans*. *PLoS ONE* 8:e54387 10.1371/journal.pone.0054387 PMC3548784 23349872

[B61] Prates R. A. Kato I. T. Ribeiro M. S. Tegos G. P. Hamblin M. R. (2011). Influence of multidrug eﬄux systems on methylene blue-mediated photodynamic inactivation of *Candida albicans*. *J. Antimicrob. Chemother.* 66 1525–1532 10.1093/jac/dkr160. 21525022 PMC3112030

[B62] Raab O. (1900). Über die Wirkung Fluoreszierender Stoffe auf Infusorien. *Z. Biol.* 39 546–546.

[B63] Rodrigues G. B. Primo F. L. Tedesco A. C. Braga G. U. (2012). In vitro photodynamic inactivation of *Cryptococcus neoformans* melanized cells with chloroaluminum phthalocyanine nanoemulsion. *Photochem. Photobiol*. 88 440–447 10.1111/j.1751-1097.2011.01055.x 22145636

[B64] Romagnoli C. Mares D. Sacchetti G. Bruni A. (1998). The photodynamic effect of 5-(4-hydroxy-1-butinyl)-22-bithienyl on dermatophytes. *Mycol. Res.* 102 1519–1524 10.1017/S0953756298006637

[B65] Sardi Jde C. Pitangui Nde S. Rodriguez-Arellanes G. Taylor M. L. Fusco-Almeida A. M. Mendes-Giannini M. J. (2014). Highlights in pathogenic fungal biofilms. *Rev. Iberoam. Micol.* 31 22–29 10.1016/j.riam.2013.09.014 24252828

[B66] Scherer H. (1841). Chemisch-physiologische untersuchungen. *Ann. Chem. Pharm.* 40 1–64 10.1002/jlac.18410400102

[B67] Schwartz S. K. Absolon K. Vernund H. (1955). Some relationships of porphyrins, X-rays, and tumours. *Univ. Minn. Med. Bull.* 27 7–8.

[B68] Sekkat N. Van Den Bergh H. Nyokong T. Lange N. (2012). Like a bolt from the blue: phthalocyanines in biomedical optics. *Molecules* 17 98–144 10.3390/molecules17010098 22198535 PMC6269082

[B69] Sharma M. Manoharlal R. Puri N. Prasad R. (2010). Antifungal curcumin induces reactive oxygen species and triggers an early apoptosis but prevents hyphae development by targeting the global repressor TUP1 in *Candida albicans*. *Biosci. Rep.* 30 391–404 10.1042/BSR20090151 20017731

[B70] Smijs T. G. Pavel S. (2011). The susceptibility of dermatophytes to photodynamic treatment with special focus on *Trichophyton rubrum*. *Photochem. Photobiol.* 87 2–13 10.1111/j.1751-1097.2010.00848.x 21114670

[B71] Smijs T. G. Schuitmaker H. J. (2003). Photodynamic inactivation of the dermatophyte *Trichophyton rubrum*. *Photochem. Photobiol.* 77 556–560 10.1562/0031-8655(2003)077<0556:PIOTDT>2.0.CO;2 12812300

[B72] Soares B. M. Alves O. A. Ferreira M. V. Amorim J. C. Sousa G. R. Silveira Lde B. (2011). Cryptococcus gattii: in vitro susceptibility to photodynamic inactivation. *Photochem. Photobiol.* 87 357–364 10.1111/j.1751-1097.2010.00868.x 21114500

[B73] Soares B. M. Da Silva D. L. Sousa G. R. Amorim J. C. De Resende M. A. Pinotti M. (2009). In vitro photodynamic inactivation of *Candida* spp. growth and adhesion to buccal epithelial cells. *J. Photochem. Photobiol*. *B* *Biol* 94 65–70 10.1016/j.jphotobiol.2008.07.013 19014890

[B74] Sotiriou E. Koussidou-Eremonti T. Chaidemenos G. Apalla Z. Ioannides D. (2010). Photodynamic therapy for distal and lateral subungual toenail onychomycosis caused by *Trichophyton rubrum*: preliminary results of a single-centre open trial. *Acta Derm. Venereol.* 90 216–217 10.2340/00015555-0811 20169321

[B75] Takahashi H. Nakajima S. Sakata I. Iizuka H. (2014). Antifungal effect of TONS504-photodynamic therapy on *Malassezia furfur*. *J. Dermatol.* 41 895–897 10.1111/1346-8138.12615 25226792

[B76] Tappeiner H. V. (1900). Über die Wirkung fluoreszierender Stoffe auf Infusorien nach Versuchen von O. *Raab. Muench. Med. Wochenschr.* 47 5–7.

[B77] Tappeiner H. V. Jodlbauer A. (1904). Über Wirkung der photodynamischen (fluoreszierenden) Stoffe auf Protozoan und Enzyme. [On the effect of photodynamic (fluorescent) substances on protozoa and enzymes]. *Dtsch. Arch. Klin. Med*. 80 427–487.

[B78] Tappeiner H. V. Jodlbauer A. (1907). *Die sensibilisierende wirkung fluorieszierender substanzer. Gesammte Untersuchungen uber die photodynamische Erscheinung*. Leipzig: FCW Vogel.

[B79] Teichert M. C. Jones J. W. Usacheva M. N. Biel M. A. (2002). Treatment of oral candidiasis with methylene blue-mediated photodynamic therapy in an immunodeficient murine model. *Oral Surg. Oral Med. Oral Pathol. Oral Radiol. Endod*. 93 155–160 10.1067/moe.2002.120051 11862203

[B80] Usuda J. Kato H. Okunaka T. Furukawa K. Tsutsui H. Yamada K. (2006). Photodynamic therapy (PDT) for lung cancers. *J. Thorac. Oncol.* 1 489–493 10.1097/01243894-200606000-00018 17409904

[B81] Weitzman I. Summerbell R. C. (1995). The dermatophytes. *Clin. Microbiol. Rev.* 8 240–259. 7621400 10.1128/cmr.8.2.240 PMC172857

[B82] White T. C. Findley K. Dawson T. L. Jr. Scheynius A. Boekhout T. Cuomo C. A. (2014). Fungi on the skin: dermatophytes and *Malassezia*. *Cold Spring Harb. Perspect. Med*. 4 a019802 10.1101/cshperspect.a019802 PMC4109575 25085959

